# Feature of catalysis on bimetallic alloys Zr with V, Mo, and Fe in the reaction of methanol oxidation

**DOI:** 10.3906/kim-2010-9

**Published:** 2021-08-27

**Authors:** Arif EFENDI, Lala MAGERRAMOVA, Adila ALIYEVA, Lyudmila KOJA-ROVA, Elmir BABAYEV

**Affiliations:** 1 Ecological Catalysis Department, Institute of Catalysis and Inorganic Chemistry, Azer-baijan National Academy of Sciences, Baku Azerbaijan; 2 Department of Physical and Colloidal Chemistry, Faculty of Chemistry, Baku State Uni-versity, Baku Azerbaijan

**Keywords:** Bimetallic catalysts, methanol oxidation, redox treatment, formaldehyde, dimethyl ether, dimethoxymethane

## Abstract

Catalytic behaviors of bimetallic catalysts-alloys of zirconium with vanadium, molybdenum, and iron was investigated in the oxidative dehydrogenation of methanol. The conditions for the formation of the catalyst’s active surface were revealed. The conversion of methanol into formaldehyde, dimethyl ether, and dimethoxymethane on bimetallic catalysts was studied. The characterization of catalysts was performed by XRD, XPS, and SEM. It was shown that the activity of samples increases after О_2_ + Н_2_ treatment and was associated with segregation of the active components of alloys (V, Mo) on the surface of catalysts and realization of their optimal oxidation state under catalysis conditions.

## 1. Introduction

Bimetallic catalysts and their industrial application on worldwide scale in petroleum reforming processes have played an important role in production of valuable hydrocarbons (lead-free gasoline, safe for environment) [1,2]. Bimetallic systems, unlike monometallic catalysts, are more active and selective, and require further investigations. Combinations of adjacent atoms in volume, the influence of the so-called “electronic factor”, preactivation conditions are the causes of increased interest.

Selective catalysts of methanol oxidation are mainly metals, metal oxides, heteropoly acids and zeolites. Methanol is a cheap and accessible raw material for the production of formaldehyde, dimethyl ether, and dimethoxymethane. 

Formaldehyde (FA) has been used as a synthesis baseline for many chemical compounds, including phenol-formaldehyde, urea formaldehyde, and melamine resin. It is produced by catalytic partial oxidation of methanol over Cu-oxide catalyst and polycrystalline Ag-catalyst [3,4]. The other route involves the oxidation of methanol over catalyst of Mo-Fe oxides [5].

Dimethyl ether (DME) has been used as an aerosol propellant, a clean alternative fuel for diesel engines, which results in lower exhaust emissions. DME can be achieved directly from CH_3_OH over different catalysts, such as γ-Al_2_O_3_;HZSM-5; Cu/ZnO/Al_2_O_3_ [6,7].

Dimethoxymethane (DMM) has been used as a solvent in the pharmaceutical and perfumery industries due for its low toxicity. It is also used as a diesel fuel additive, has a high cetane number (˃ 80). Catalysts such as rhenium oxides [8], MoOx/TiO_2 _[9], modified with VO_x_/TS-1 [10], and V-complex oxides [11,12] showed a good performance in the selective oxidation of methanol. Vanadium oxides, as highly efficiently catalysts, are widely applied in various catalytic reactions, such as the dehydrogenation hydrocarbons [13]. All these catalysts are complex systems, the study of activities of which is difficult due to influence of numerous factors. However, in bimetallic catalyst systems, introduction of the second component creates the possibility of varying catalytic properties in a wide range [14].

Vanadium oxide-based catalysts are well known and extensively employed in industry for the gas phase heterogeneous oxidation of hydrocarbons and for the oxidative dehydrogenation of С_2_-С_4_ alkanes and alcohols [15]. During the oxidation of methanol, as it was described in the previous studies [15,16], the catalytic activity of supported vanadium catalysts decreased in the following trend: VO_x_/ZrO_2_>VO_x_/(Al,Zr-oxides) > VO_x_/Al_2_O_3 _and was dependent on the nature of the support. Formaldehyde was the main reaction product on catalysts supported on Zr-containing oxides (which can be related to a low amount of acid sites). However, dimethyl ether was mainly observed on alumina-supported vanadium oxide catalysts (which is linked to the presence of acidic sites on the surface of the catalyst, as determined by TPD-NH_3_). Further studies showed [17–23] that following factors had a great impact on the catalytic behavior of vanadium-supported catalysts: (i) the nature of V^ n+^─ O species, i.e., coordination, aggregation, and oxidation state of V-species; (ii) redox properties of the catalysts, i.e., the V^5+^/V^4+^ ratio in reaction, (iii) acid–base character of the catalysts (as well as the support), that determines the selectivity of reaction products.

It is well-known that the support of ZrO_2_ has bifunctional redox properties of both an acid and a base, as well as possessing a great thermal stability. By modifying the aluminum oxide support by adding ZrO_2_, it was possible to obtain a more efficient catalyst for the dehydrogenation of ethane and methanol [15,17]. The study showed that both ZrO_2_ [18], and Al_2_O_3_-ZrO_2_ [19,20] are appropriate supports for the synthesis of highly dispersed VOx-species [20,24,25]. As shown by XRD patterns, supported vanadium oxide VOx can exist on these supports in the form of isolated tetrahedral monovanadates, one- or two-dimensional polyvanadate domains, or bulk V_2_O_5 _crystallites [21].

Oxygen particles that bind surface vanadium oxide particles to the support can play an important role in catalyst activity. Thus, the VO↔ bond on the support mainly determines its catalytic characteristics. In the vanadium oxide catalysts support on pure ZrO_2_, the presence of both monoclinic and tetragonal crystallites of ZrO_2_ and V_2_O_5_ (in the sample with the highest V load) was found. Moreover, the presence of mixed oxides Zr-V, such as ZrV_2_O_7_, cannot be excluded [15,22].

Quantum-chemical evaluations of the V_2_O_5 _structure revealed two types of crystal planes [23]. On the (100) and (001) planes, the cleavage leaves coordination unsaturated vanadium and oxygen ions, which develop Bronsted acid-base interactions with the reacting molecules. The equilibration of gas phase oxygen with vanadium oxides leads to the formation of an internal defect structure of V_2_O_5_, consisting of oxygen vacancies. In the previous studies [24–28] the unique physical and chemical characteristics of supported vanadium catalysts in comparison with other catalysts supported by metal oxides for hydrocarbon oxidation reactions were discussed, covering the required number of surface vanadium centers, the effect of metal oxide additives, the effect of acidic and basic centers on the surface, the effect of preparation methods and the effect of a specific phase of the oxide substrate. 

The acid-base character of the metal oxide support affects the dispersion of vanadium on the surface of the support, as well as the nature of the vanadium species. The reducibility and structure of surface vanadium oxide particles and the acid-base character of catalysts, in addition to their catalytic properties in the oxidative dehydrogenation of alkanes, strongly depend on the metal oxide used as a support and the vanadium content of the V^5+^ tetrahedral species [19]. 

Molybdenum catalysts are less active than vanadium ones, however, catalytic properties increase on zirconium oxide ZrО_2_. The impact of the support structure, as well as acid sites and dispersion were studied [29]. In order to enhance the activity of MoO_3_/ZrO_2_, preliminary treatment with hydrogen, the introduction of vanadium as a second component was proposed. The activity of the Mo^5+^ ion in catalytic processes has been confirmed in a number of studies. Thus, in a study by C. Ranga et al. [30] an evaluation of the surface composition and state of Mo surface particles was carried out on samples of MoO_3_/ZrO_2_ catalysts, fresh and reduced with H_2_/CH_4_ (XPS). The fresh sample contained only Mo^6+^ particles, and moderate reduction of the MoO_3_ sample at T 623 K resulted in the presence of Mo^5+^ particles along with Mo^6+^, as well as Mo^4+^. The presence of Mo^5+^ particles in reduced samples, according to the authors, indicated the creation of active defects in the structure of the starting oxide, which leaded to an increasing activity. It was also reported that Mo^5+^ particles appeared as a result of the reduction of MoO_3_ to MoO_ 3- x_. Interesting studies of the selective oxidation of methanol in DMM were carried out on the V_2_O_5_–MoO_3_/γ-Al_2_O_3_ catalysts by Yali Meng et al. [31]. These catalysts exhibit better DMM yield characteristics than the corresponding samples containing only V and Mo particles, even at relatively low temperatures. By adjusting the V content, catalysts were investigated and explained by the synergistic effect of mixed oxides of V and Mo that are present on the catalyst surface. The redox cycle of V-O-Mo oxides can be completed through the transfer of electrons between lattice oxygen and metal cations. V species exhibit superior performance in adsorption and activation of oxygen gas, as well as an increased ability to reduce lattice oxygen and suppress Mo particle aggregation. 14V_2_O_5_–14MoO_3_/Al_2_O_3 _catalyst with an optimized number of redox and acid sites was obtained, which have demonstrated a methanol conversion of 54% with a DMM selectivity of 92% at 393 K. 

All considered vanadium and molybdenum catalysts belong to supported oxides, with different methods of preparation. The correlation of their activity with the type of support, acidity, structure, number of redox centers was examined. However, research studies on alloys has not been conducted. We tried to carry out such evaluation of oxidative dehydrogenation of methanol, which appeared to be overly sensitive to the effects of various factors. Alloys can be considered the models, on which conditions, affecting for the formation of the active surface can be studied [32,33]. 

## 2. Experimental 

### 2.1. Chemicals and reagents

All chemicals and reagents were of analytical grade and used without any further treatment. The following materials were used to prepare alloy samples: molybdenum (powder, TC 48-19-316-80, purity ~99.5%; melting temperature 2.89 K), Vanadium (ingots, ТС48-4-272-73, purity ~99.4%; melting temperature 2.160 K), Zirconium (ingots, manufacturer YWBL-WH, purity ~99.4%; melting temperature 2.128 K), iron (powder, OCHV. Ru., purity~99.8%; melting temperature 1.812 K), absolute methanol (99.9%; AzMeCo).

### 2.2. Catalyst preparation 

Initial samples of Zr alloys with V, Mo, and Fe of various compositions were prepared by fusing metal weighed portions in an arc furnace based on the ratio of atomic weights, for example: intermetallic compound ZrMo_2_: A_r _(Zr) = 91.224; A_r _(Mo) = 95.94.     

M_r__ _(ZrMo_2_) = 91.224 + 2 × 95.94 = 91.224 + 191.88 = 283.104.        

Molybdenum content     191.88: 283.1 = 67.8 wt.% Mo.       

Zirconium content         91.224: 283.1 = 32.2 wt.% Zr.

To obtain 20 g of ZrMo_2_ alloy: 20.0 × 0.678 = 13.6 g Mo and 6.4 g Zr are needed. 

After melting, ZrMo_2 _ingot weighing – 17.6 g; ∆ = –2.4 g was obtained.

Following samples of catalysts were obtained and showed in Table 1: 

**Table 1:** Compositions of the obtained catalysts.

№	Catalyst	Sample composition, (% wt).	Amount, g	Weight, g
1	М_r_ (ZrMо2) = 283.10	ZrMо_2_ (67.8 Mo )	6.4 Zr + 13.6 Mo	20.0 ZrMо2
2	М_r_ (ZrMо) = 187.16	ZrMo (51.3 Мо)	7.3 Zr + 7.7 Mo	15.0 ZrMo
3	M_r_ (ZrMo 0.5) =139.22	ZrMo 0.5 (34.48 Mo)	9.82 Zr + 5.18 Mo	15.0 ZrMo 0.5
4	M_r_ (ZrV) = 142.16	ZrV (35.83 V)	12.84 Zr + 7.16 V	20.0 ZrV
5	M_r_ (Zr 0.6) = 121.79	ZrV0.6 (25.09 V)	14.98 Zr + 5.02 V	20.0 ZrV0.6
6	M_r_ (Zr 0.3) = 105.6	ZrV0.3 (14.35 V)	17.13 Zr + 2.87 V	20.0 ZrV0.3
7	M_r_ (Vfe 0.6) = 84.45	VFe 0.6 (39.68 Fe)	12.06 V + 7.94 Fe	20.0 VFe 0.6
8	M_r_ (VFe 0.3) = 67.70	VFe 0.3 (24.76 Fe)	14.06 V+ 4.94 Fe	20.0 VFe 0.3
9	M_r_ (VFe 0.2 ) = 62.11	VFe 0.2 ( 17.98 Fe)	16.4 V + 3.6 Fe	20.0 VFe 0.2

ZrV_0.3 _; Zr_0.6_ ; ZrV; ZrMo_0.5_; ZrMo ; ZrMo_2_ , VFe_0.2_; VFe_0.3_ ; VFe_0.6.__ _

The metal weighed portions were preliminarily pressed, then fused in an arc furnace in an atmosphere of high purity helium. To obtain homogeneous uniform alloys the ingots were re-melted 2–3 times until uniform homogeneity was obtained. Samples of catalysts, then grounded to a particle size of 0.06–0.10 mesh, were mixed with crushed glass, quartz, and then placed into a tubular quartz reactor. The preliminary oxidative treatment of the catalysts was carried out in the air stream at T 873 K 3–4 h, and reducing treatment was carried out in the hydrogen stream at T 873 K for 1 h.

### 2.3. Characterization

Changes in the activity of catalysts are associated with phase changes in the surface layer, thus, X-ray diffraction analysis (XRD) of catalysts were carried out before and after О_2_ + Н_2_-treatment on Rigaku Mini Flex 600 X-ray diffractometer (k¼ 1.54060 Ǻ) using Ni- filtered CuKα radiation, powder diffractometer Bruker “D2 Phaser “and DRON-2 with CuKα radiation.

Changes were observed not only in the composition of the surface layer, but also in the valence state of the active components of alloys (V, Mo), as indicated by the results of X-ray photoelectron spectroscopy (XPS), which was carried out on V1EE-15 and ADES-400 spectrometer. The specific surface area of ​​the catalyst samples was determined using Thermo Scientific SURFER. The scanning electron microscope (SEM) HITACHI S-3400N was used for the observation of specimen surfaces. 

### 2.4. Experimental part

The examination of the catalytic activity of alloy samples was carried out in a quartz reactor at pulsed and flow modes (weighed number of catalysts for a pulse unit - 0.2 g; for a flow-through unit - 0.3 g). On a pulse installation carrier gas, helium from the chromatograph system entered a quartz reactor, into which, at T 473–673 K, samples (methanol) are injected using a micro syringe. The experiment was carried out both with an air supply (through a dispenser) and without air. After reactor, the products, together with helium carrier-gas, enter to the separation column of the chromatograph (GC); filled with Porapak-Q (3m), then flow into the thermal conductivity detector and further to TSVET-500 chromatograph recorder. The column temperature was maintained within the range of 413–418 K.      

On a pulsed installation, the reactor was placed in an electrically heated furnace. The reaction temperature has been controlled by a thermocouple (TXK), placed inside the catalytic reactor at the level of the catalyst bed. Additional conditions of the experiment will be explained in section 3.2.

On a flow-through unit, alcohol vapors, taken up by nitrogen, passed through the distillation plates of the saturator, then entered the mixer, where purified, dried oxygen was supplied. The mixer was placed in the thermostat, where a constant temperature of 423 K was maintained. The gas mixture was fed into a quartz reactor heated in a thermocouple controlled furnace. The off-gas measurement (and contact time) was carried out using a GSB-400 drum gas meter. The time to reach the stationary mode was determined according to the constant composition of the outgoing reaction mixture; average time was ~ 20–30 min. When the stationary regime was reached, the gas flow was switched on a six-way valve at the outlet of the reactor, staying at position 2 (sampling). At the same time, the value of the reactor temperature, flow rate, O_2_/N_2_ ratio, alcohol flow temperature at the outlet from the saturator, partial pressures Psp; Po_2_; alcohol/O_2_ ratio were measured and noted. 

Samples of alloys did not show catalytic activity in the oxidation of methanol until T 673––773 K. However, after О_2_-treatment with air at Т 873 K for 3 h an increase of activity was observed. After reduction treatment in a stream of hydrogen at Т 873 K for 1 h, the activity of the catalyst increased sharply (a scheme of a flow-through installation is given in the appendix in Supplemental Figure 1S).

## 3. Results and discussion

### 3.1. Catalysts characterization

**Figure 1:**
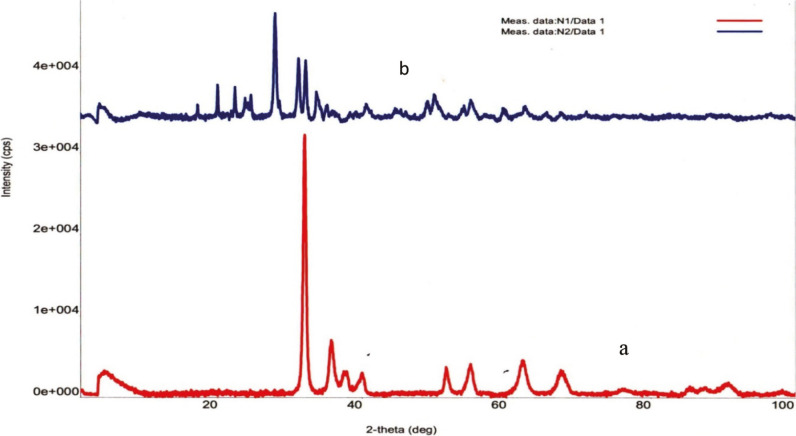
XRD patterns of catalyst based on an alloy ZrV0.3: a) initial ZrV0.3; b) after O2- treatment, T 873 K, 3 h.

The initial samples of alloys did not show catalytic activity in the oxidation of methanol until T 773 K, however, at the temperature above T 773 K the formation of products of deep oxidation and decomposition was observed. Treatment in vacuum, flow of helium, and hydrogen did not lead to any change in the activity. Only after oxidative treatment with air at T 873 K for 1 h, the catalysts began to show activity, and after 3-h air treatment, the activity of the samples increased and stabilized. It is well known that when an alloy encounters any adsorbate, its surface becomes enriched with a metal that forms a stronger bond with this adsorbate. In terms of oxygen, zirconium possesses a high affinity for it, based on thermodynamic calculations (∆G_ZrO2 _= –1039 kJ/mol; ∆G_V2O4 _= –334 kJ/mol; ∆G_ V2O5 _= –365.6 kJ/mol), where ∆G ─ Gibbs function. X-ray diffraction analysis (XRD) of the samples showed that on the catalyst surface, zirconium is mainly present in the oxidized phase (see Figure 1a).

The X-ray diffraction pattern of the initial ZrV_0.3_ sample, showed peaks at 2θ = 32.0°; 35.7^o^; 51.59°; 62.3^o^; 67.5^о^, which could be associated with the presence of both monoclinic and tetragonal structures of ZrO_2_ (JCPDS: 37-1484, 17-923). Peaks at 2θ = 32.0; 35.8; 40.1; 54.9 were associated with the presence of metallic zirconium (JCPDS: 5-665). Thus, following products were found on the surface of the initial sample: mainly metallic Zr, m-ZrO_2_; t-ZrO_2_. Vanadium and vanadium oxides were absent. After О_2_-treatment with air (Т 873 K, 3 h), the activity of the catalyst increases, which is associated with phase changes in the surface layer (Figure 1b; Supplemental Tables 1 and 2). 

**Table 2:** X-ray photoelectron spectroscopy of alloy ZrMo2 after treatments.

№	Terms of processing	Mo content inoxidation state, %			Mo/Zr	I 0,1SI Zr 3d + I Mo 3d
		VI	IV	0		
1	Initial ZrMo2	49	21	30	1.1	0.6
2	Vacuum, T 673 K	9	5	86	1.1	0.3
3	Air, T 873 K, 3 h	100	0	0	1.4	0.6
4	Hydrogen, 873 K, 1 h	22	-	78	1.4	0.6
5	Air, T 873 K, 3 h + Hydrogen, T 873 K,1 h + Catalysis	65	35	-	1.4	0.6

The peaks that appeared on the diffractogram are associated with the presence of mixed oxides - orthorhombic m-V_2_O_5_ on the catalyst surface (at 2θ = 20.3; 26.2; 31.2; 34.3; 47.3; 50.5; JCPDS: 41-1426), tetragonal t-V_2_O_5_ (at 2θ = 18.6; 23.6; 30.2; 35.5; 44.5; 50.7; 53.5; 57.4; 61.0; JCPDS: 79- 1976), V_2_O_3_ (at 2θ = 23.6; 32.7; 37.6; 41.8; 53.9 JCPDS: 34-0187). The 2θ values ​​for VO_2_, V_4_O_9_ oxides are remarkably close, that lead to the failure to identify them (JCPDS: 81-2392; 43-1051).          

**Figure 2:**
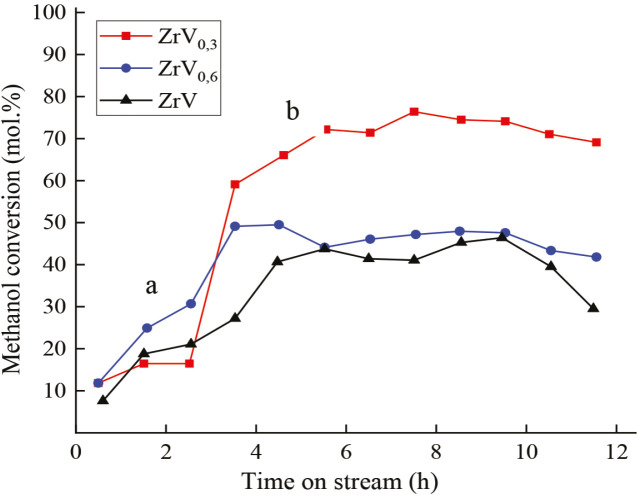
Variation of methanol conversion on catalysts Zr–V: a) after O2-treatment; b) after H2-treatment, reaction temperature 473 K.

**Figure 3:**
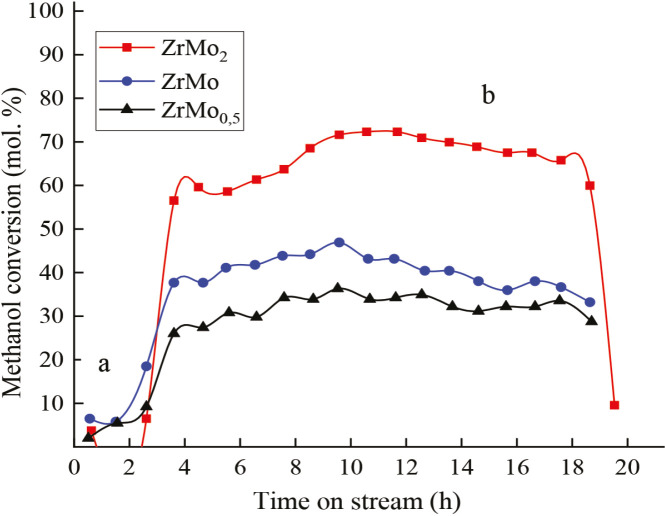
Variation of methanol conversion on catalysts Zr-Mo: after O2-treatment; b) after H2-treatment, reaction temperature – 473 K.

**Figure 4:**
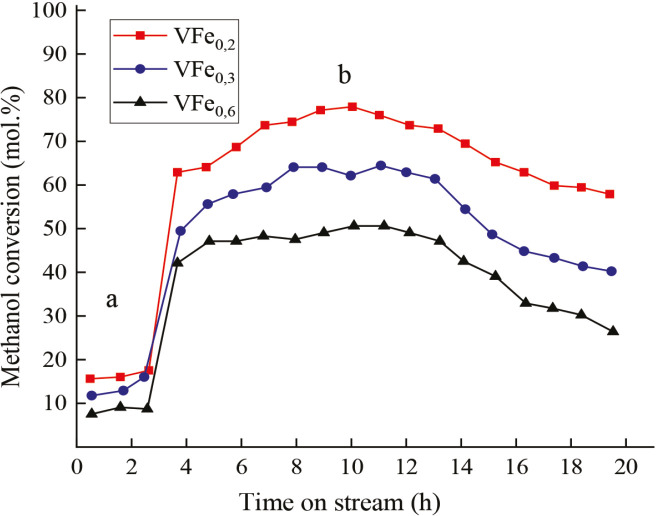
Variation of methanol conversion on catalysts V–Fe; a) after O2-treatment; b) after H2-treatment, reaction temperature – 473 K.

Although V_2_O_5_ and V_2_O_3_ oxides were present on the surface of the ZrV_0.3_ catalyst after О_2_-treatment, the activity of the sample in the oxidation of methanol increased insignificantly (the conversion of СН_3_ОН at T 473K was 9.6%, and at T 573K- 46.2%). Only after H_2_-treatment, the activity of the catalysts increases drastically, which can be seen from Figures 2–4 for Zr-V, Zr-Mo and V-Fe alloys.

The study of methanol conversion, presented in these figures, was performed in a pulsed mode. Carrier gas, helium, from the chromatograph system entered the reactor, and then moved to separating column and to the thermal conductivity detector. Methanol was introduced with a microsyringe (1–2 μL) at a temperature range 473–573 K, in the presence of oxygen and without it. The experiments were carried out for a long time (10–20 h) in order to check the activity of the catalyst samples and to select the most active ones. The reaction products contained FA, DME, DMM, and MF. The data of these experiments were confirmed later when carrying out the reaction in a flow-through unit explained in section 3.2. 

**Figure 5:**
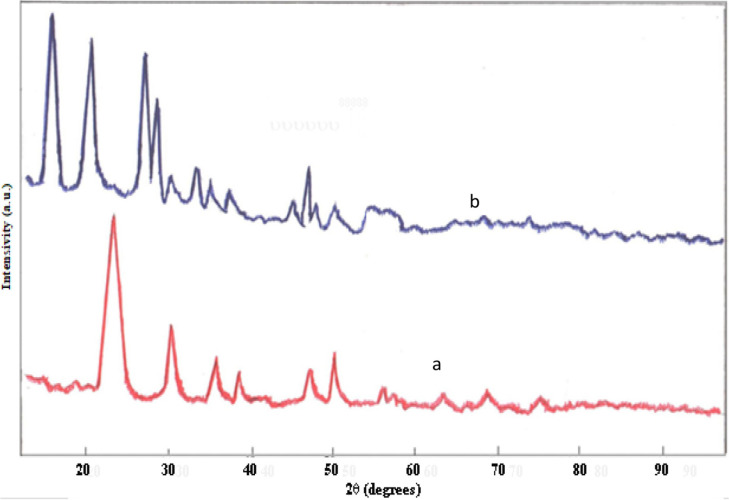
XRD patterns of catalyst based on an alloy ZrMo2; a) after O2-treatment, 873K, 3h, b) after H2-treatment, 873 K, 1 h.

If vanadium was absent on the surface of the initial ZrV_0.3_ sample, after О_2_-treatment it emerged to the surface, enriched of the surface layer with vanadium (segregation) and later oxidized. The V/Zr ratio in the surface layer increased. ZrO_2_ oxide remained at the bottom of the catalyst as a support. Deep phase changes in the surface layer were also observed for the ZrMo_2_ catalyst after О_2_-treatment (Т873 K, 3 h), following by Н_2_-treatment (Т873 K, 1 h), as it is described at Figures 5.

Mainly the phase of zirconium molybdate Zr (MoO_4_)_2_ is formed on the surface of the ZrMo_2_ catalyst after О_2_-treatment. The experimental data and the data of the reference diffraction patterns coincide. In addition, there were slightly shifted peaks corresponding to MoO_3_; no noticeable increase in the activity of the catalyst was observed. However, after Н_2_-treatment (Т 873 K, 1 h), the activity of the catalyst increased sharply, and at a reaction temperature of 473 K the methanol conversion was 56.5% - Figure 3. The increase in activity was associated with phase changes on the sample surface (Supplementary Tables 3 and 4).

After О_2_-treatment it was possible to identify the Zr (MoO_4_ **)**_2_ phase only on the ZrMo_2_ intermetallic compound. However, after H_2_ – processing, the largest peaks at 2θ = 15.646; 21.034 (d = 5.642Ǻ, 4, 231Ǻ) did not correspond to any of the oxide compounds of molybdenum. Peaks at 2θ = 22.6; 24.7; 26.2 were close to nonstoichiometric i-Mo_4_O_11_ oxides (d = 3.93Ǻ, 3.60Ǻ; 3.41Ǻ; card 13-142), with 2θ = 24.84; 26.41; 27.25 - corresponding to Mo_9_O_26_ (d = 4.00Ǻ; 3.75Ǻ; 3.48Ǻ; card 12-753). Formation of other nonstoichiometric MoOx oxides are possible. Peaks observed at 2θ = 56.46; 68.65; 76.9; refer to recovered, partially metallic molybdenum (JCPDS: 01-1208). According to the diffraction pattern, the surface composition is characterized by H_6_Mo_2_O_11_Zr formula. 

The nonstoichiometric composition of ZrMO_2_ catalyst surface after О_2 _+ Н_2_-treatment led to an increase in the activity of the sample at a reaction temperature of 473 K. Figure 5 shows that in the course of catalysis, the conversion of methanol increased from 56.5% to 72.6% due to the further formation of an active surface under conditions of aerobic oxidation of methanol. 

**Figure 6:**
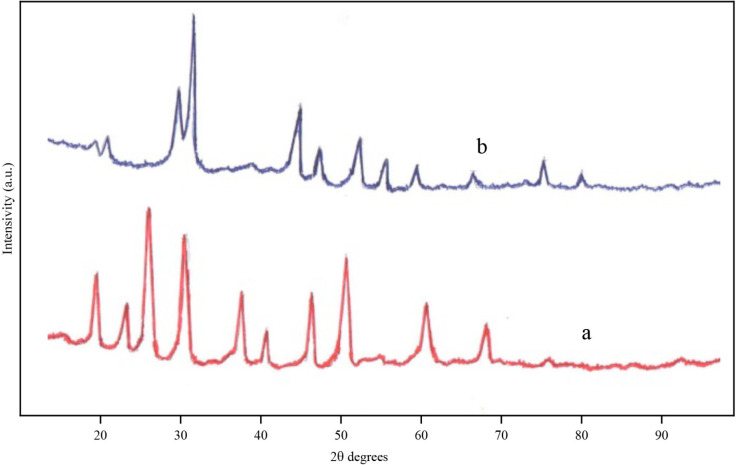
XRD patterns of catalyst based on an alloy VFe0,2; a) after O2-treatment, 873 K, 3 h; b) after H2-treatment, 873 K, 1 h.

Ability of the surface to absorb hydrogen with the formation of hydride phases plays an important role in formation of the active surface of alloys. While zirconium acts as a hydride-forming component in its alloys, vanadium acts as such a component in the VFe alloy. The ability of vanadium to form numerous compounds with both oxygen and hydrogen makes it unique. Figure 4 shows the change in the catalytic activity of the VFe_0.2_ alloy after О_2_- treatment (Т 873 K, 3 h) and Н_2_-treatment (Т 873 K, 1 h). The observed phase changes on the contact surface are shown in Figure 6 (Supplemental Table 5).

On the surface of the initial VFe_0.2_ alloy, as can be seen from Supplemental Table 5, mainly metal-vanadium (at 2θ = 41.3; 42.2; 62.6), and insignificantly - V_2_O_3_ oxide (at 2θ = 32, 7; 35.1) are present. No iron phase was detected.

As a result of oxidative treatment with air (T 873 K, 3 h), the formation orthorhombic and tetragonal oxide V_2_O_5_ phase is observed on the catalyst surface (at 2θ = 20.6; 24.6; 27.0; 32.5; 50.2; Figure 6). Formation of the VO_2_ phase (2θ = 26.3; 37.1; 42.0; 56.5 JCPDS: 43-1051), other VO_x_ oxides, mixed oxides, which are formed in large quantities by vanadium with oxygen, are possible as well. However, the activity of the VFe_0.2_ catalyst remains at a low level. Only after H_2_-treatment (T 873 K, 1 h), drastic increase in the activity of the sample observed (Figure 4), which is obviously associated not only with a change in the phase composition of the surface, but also with the valence state of the components. If after О_2_-treatment there was no iron phase on the sample surface, then after Н_2_-treatment there is a segregation of iron on the surface, its oxidation, the appearance of new peaks of α-Fe_2_O_3_, FeO mixed with Fe_3_O_4,_ possibly mixed with vanadium Fe-V-O of various compositions, the phases which are almost impossible to establish **.**

According to thermodynamic data, iron (II), and vanadium (III, IV, V) oxides have close Gibbs energies (∆G^o^_298_ (VO) = –402.6 kJ/mol; ∆G_VO2 _= –665.0; ∆G _FeO_ = –244.3; ∆G_Fe2O3_ = –740.3; ∆G_V2O3_ = –1139.4; ∆G_V2O5_ = –1421.2;), and the formation of such oxides is not excluded. Formation of nonstoichiometric oxides V_4_O_9_, V_3_O_7,_ V_2_O_4_ of different structures after Н_2_-treatment is also possible. In homogeneous composition of the surface, as well as the presence of anionic vacancies, changes in the valence state of the components led to an increase in the activity of VFe_0.2_ after О_2_ + Н_2_-treatment.

**Figure 7:**
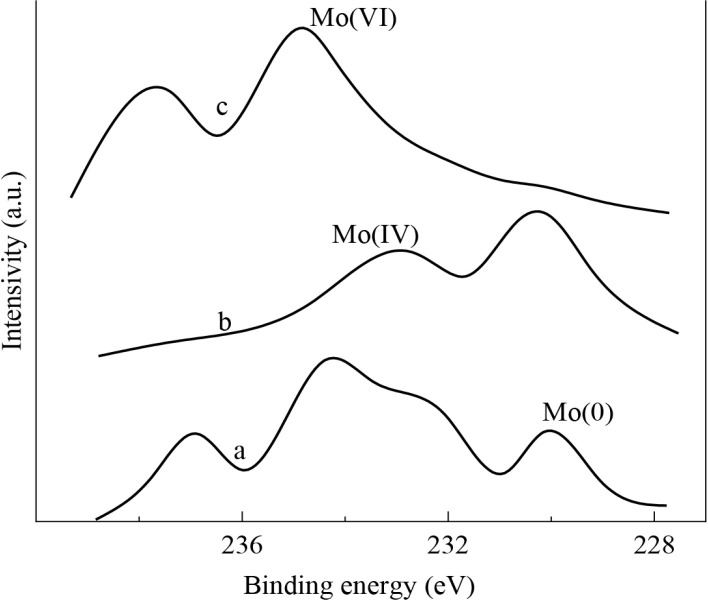
X-ray photoelectron spectra of catalyst ZrMo2. a) Initial. b) After H2-treatment, 873 K, 1 h. c) After O2-treatment, 873K, 3 h.

The analysis of the alloys surface and the state of the components of the alloys before and after the О_2_- and Н_2_-treatment were carried out by X-ray photoelectron spectroscopy (XPS) method on a VIEE-15 and ADES-400 spectrometer. Figure 7 shows the X-ray spectra of the ZrMo_2 _intermetallic compound depending on the processing conditions. 

On the initial surface of the sample, molybdenum is present in several valence states - in the form of an oxide, in the form of a compound in an intermediate oxidation state, and in a metallic state. Vacuum heat treatment of the sample surface by bombardment with Ar^+^ ions leads to an increase in the fraction of Mo in the zero-valent state. The binding energy of Mo 3d½ in the zero-valence state is 228.1 ± 0.1 eV. Oxidation treatment with air at T 873 K caused significant changes, mainly in the valence state of molybdenum. With an increase in the oxidation temperature, molybdenum completely transforms into a hexavalent state, the binding energy is 234.2 ± 0.1 eV, and the Mo/Zr ratio in the surface layer increases by 1.4 times. The reduction of the oxidized sample with hydrogen (T 873 K, 1 h) leads to the appearance of Mo ions in the surface layer in an intermediate oxidation state: Mo (0); Mo (IV); Mo (VI) - Table 2. 

The shape and position of the Mo 3d signal undergoes the greatest changes depending on the processing conditions. In the ZrMo_2_ sample, it was possible to identify three oxidation states of molybdenum: Mo^6+^, Mo^4+^, and Mo^0^, which correspond to the binding energy of 234.2 ± 0.3; 230.3 ± 0.2 and 228.0 ± 0.2 eV. As can be seen from Figure 3, the activity of the ZrMo_2_ catalyst after О_2_-treatment increases insignificantly, although the presence of Zr (Mo_4_)_2_ and MoO_3_ phases, i.e., Mo^6+^ ion, was established on the surface. However, after H_2_-treatment, the activity of the test sample increases significantly. According to XPS data, the presence of Mo^4+^ and Mo^0 ^ions was detected on the surface, which is consistent with XRD analysis data. In the course of catalysis, under conditions of aerobic oxidation of methanol, the activity of the catalyst continues to increase due to the further formation of the active surface. The process continues until the optimum oxidation state of Mo is achieved. Such a catalyst worked in catalysis with high efficiency for more than 20 hours, after which there was a tendency for a gradual decrease in activity. Subsequent additional oxidation in a stream of air at T 873 K, 1 h. leads to a significant decrease in activity – Figure 3. This confirms the relationship between the oxidation state of molybdenum and the activity of the system. The optimal oxidation state of the catalyst in this process is obviously the presence of the Mo^5+^ ion. The activity of the Mo^5+^ ion in catalytic processes has been confirmed in a number of studies. Thus, in a study by C. Ranga et al. [30], an evaluation of the surface composition and state of Mo surface particles was carried out on samples of MoO_3_/ZrO_2_ catalysts, fresh and reduced with H_2_/CH_4_ (XPS). The fresh sample contained only Mo^6+^ particles, and moderate reduction of the MoO_3_ sample at T 623 K resulted in the presence of Mo^5+^ particles along with Mo^6+^, as well as Mo^4+^. The binding energies were calculated for Mo^6+^, Mo^5+^, Mo^4+^ (232.0–232.4, 231.0–231.6, and 229.1–229.7 eV, respectively. The presence of Mo^5+^ particles in reduced samples, according to the authors, indicated the creation of active defects in the structure of the starting oxide, which leaded to an increasing activity. It was also reported that Mo^5+^ particles appeared as a result of the reduction of MoO_3_ to MoO_3-x._

О_2_ + Н_2_-treatment led to changes not only in the composition of the surface layer, but also to changes in the valence state of the active components of the alloy - V, Mo, as evidenced by the results of XPS analyzes and another - VFe_ 0,2_ alloy catalyst. Formation of VOx; VO_2-x_; V_2_O_3-x_; V_2_O_5-x_; V_3_O_7_; V_4_O_9_; Fe-Vx-Oy oxides and other compounds of nonstoichiometric composition on the sample surface after О_2_ + Н_2_ - treatment, the presence of anionic vacancies, the presence of defects – all contributed to the creation of active centers of two V^4+^ and V^5+^ ions on the catalyst surface (two peaks 2p 3/2 at 515.3 eV and 516.9 eV discovered by XPS belong to vanadium). The presence of an active V^4+^ ↔ V^5+^ site on the contact surface promoted an increase in the activity of VFe _0.2 _catalyst in the methanol oxidation reaction. 

**Figure 8:**
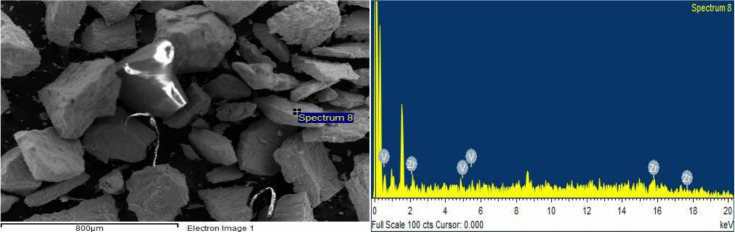
SEM image (a) and EDX spectrum (b) of ZrV0.3 catalyst after O2-treatment (873 K, 3 h).

**Figure 9:**
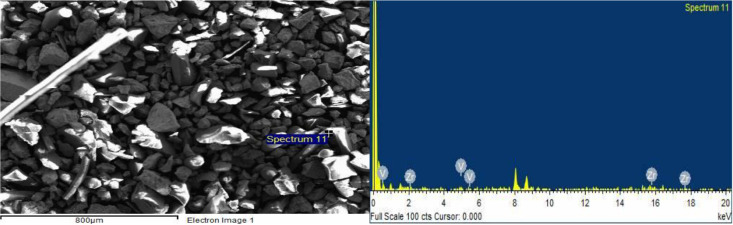
SEM image (a) and EDX spectrum (b) of ZrV0.3 catalyst after H2-treatment (873 K, 1 h).

**Figure 10:**
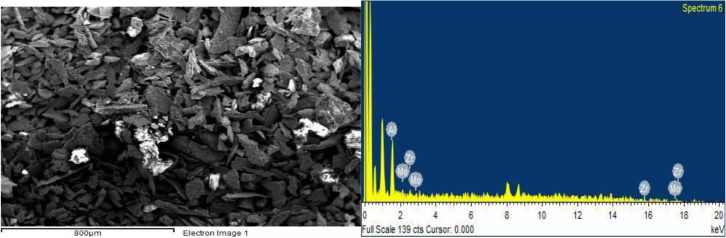
SEM image (a) and EDX spectrum (b) of ZrMo2 catalyst after O2-treatment (T 873 K, 3 h).

**Figure 11:**
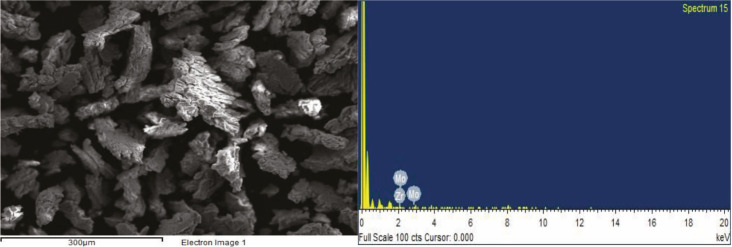
SEM image (a) and EDX spectrum (b) of ZrMo2 catalyst after H2-treatment (T 873 K, 1 h).

The surface of ZrV_0.3_ and ZrMo_2 _catalysts before and after О_2_ + Н_2_ - treatment was also investigated by the SEM method and is explained in Figures 8 (a, b), 9 (a, b) and Figures 10 (a, b), 11 (a, b).

As seen from the micrographs (SEM image), as a result of О_2_- treatment, the segregation of catalyst active components (V, Mo) on the surface increases, and accordingly increases the activity. After Н_2_-treatment, according to EDX spectrum, the V/Zr ratio and Mo/Zr slightly decreases, however, the activity of the samples increases. 

Previous researches have shown that the effectiveness of catalysts is determined mainly by the presence of active sites on its surface associated with the influence of acid-base sites, with synergistic effect, with the presence of charged particles such as Mo^5+^. Our results confirm the influence of these factors, adding that the О_2_- and Н_2_-treatment is an effective method for the formation of active centers on the surface, and under certain conditions can affect the yield and selectivity of the obtained oxidation products. In this case, alloys can be considered a model for studying the conditions for the formation of active centers on the surface of contacts.

### 3.2. Oxidative dehydrogenation of methanol

Oxidation-reduction treatment leaded to qualitative changes in the surface composition due to the segregation of the active components of alloys (Mo, V) of variable valence on the catalyst surface, and then to the implementation of the optimal degree of their oxidation under catalysis conditions. The increase in activity was associated with the appearance of Mo and V in these catalysts in an intermediate oxidation state.

The analysis of the catalytic activity of ZrV_0.3_, ZrMo_2_,and VFe_0.2 _samples was carried out on a flow-through unit, corresponding diagram is given in the appendix (Supplemental Figure 1S). Weighed portions of catalysts in an amount of 0.3 g were crushed, mixed with crushed quartz in a ratio of 1:10, then, were loaded into a tubular quartz reactor. Following О_2_ and Н_2_–treatment, reactor was places in gas thermostat, where a constant temperature was maintained. Analysis of primary samples was performed (for a description, see Section 2.3). Alcohol was fed into the reaction using nitrogen in a bubbling manner through a saturator placed in a liquid thermostat at a rate of 1.5–1.8 L/h. With an increase in the thermostat temperature, the partial pressure of alcohol increased. Oxygen was also supplied to the reactor from the cylinder, after passing purification system (alcohol: O_2_ ratio = 1 ÷ 5). GHSV - gas hourly space velocity in terms of the catalyst volume (V cat. ~ 1.5 cm^3^) was in the range of 0.5–1.2 h^–1^. After reaching the stationary mode, samples were obtained on a six-way valve, directly into the chromatograph. The volume of the exiting gas mixture was recorded on a gas meter at the outlet of the system. The reaction was performed at the temperature range 423–623 K. The most active samples were ZrV_0.3_, ZrMo_2 _and VFe_0.2_ after H_2_- treatment (T 873 K, 1 h) in the oxidative dehydrogenation of methanol at T 473 K. These include samples with a lower content of the active component, except for the ZrMo_2_ intermetallic compound. 

**Figure 12:**
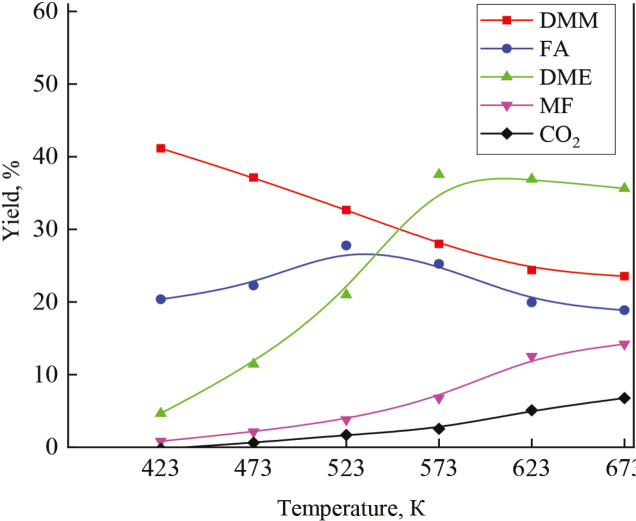
Variation of reaction yield with the reaction temperature on ZrV0.3 catalyst after O2 + H2-treatment.

The impact of temperature on the methanol oxidation reaction final products yield on the ZrV_0.3 _catalyst is shown in Figure 12. With an increase in the reaction temperature, the yield of DMM – decreases, but the yield of DME increases; meanwhile, the yield of FA passes through a maximum in the temperature range 423–473 K. When decreasing the temperature to 423 K, the DMM yield increases. A similar picture is observed for other catalysts, such as ZrMo_2_; VFe_0.2_ - Table 3.

**Table 3:** Changes in the activity of ZrV0.3 ZrMo2 and VFe 0.2 catalysts after О2- and Н2-treatment.

№	Conditions	T, K	Reaction yield, mol, %					Selec.DMM mol.%	Conver. mol. %
			СО2	FA	DME	DММ	МF		
1.	Initial ZrV0,3	673	27.3	-	-	-	-	-	27.3
2.	ZrV0,3Air, Т 873 K, 3h	423	-	-	2.0	-	-	-	2.0
		473	-	9.6	-	-	-	-	9.6
		523	-	19.6	-	-	-	-	19.6
		573	1.1	45.2	-	-	-	-	46.2
		623	2.3	69.8	-	-	-	-	73.1
3.	ZrV0,3Air, Т 873 K, 3hH2-,Т 873 K, 1h	423	0.1	20.3	4.8	41.0	1.0	61.0	67.2
		473	0.8	226	11.5	38.0	2.4	50.4	75.3
		523	2.0	28.2	20.8	33.4	3.9	37.8	88.3
		573	3.0	25.7	38.0	28.6	6.7	28.6	100
		623	5.2	20.2	37.3	25.0	12.3	25.0	100
4	Initial ZrMo2	673	20.5	-	-	-	-	-	20.5
5.	ZrMo2Air, Т 873 K, 3h	423	-	weak	-	-	-	-	
		473	-	6.0	-	-	-	-	6.0
		523	-	21.6	-	-	-	-	21.6
		573	0.7	42.2	-	-	-	-	42.2
		623	2.2	51.9	-	-	-	-	539
6.	ZrMo2Air, Т 873 K, 3hH2-,Т 873 K, 1h	423	0.1	18.1	5.1	39.3	0.8	62.0	63,4
		473	1.9	22.0	10.2	37.1	1.4	51.1	72.6
		523	2.2	27.0	21.3	32.7	3.1	37.9	86.3
		573	3.0	29.7	36.8	26.0	4.5	26.4	98.6
		623	5.1	25.0	38.3	24.2	7.4	24.2	100
7	Initial VFe0,2	673	21.4	-	-	-	-	-	21.4
8	VFe0,2Air, Т 873 K, 3h	423	-	-	-	-	-	-	14.7
		473	-	15.2	-	-	-	-	15.2
		523	-	22.9	-	-	-	-	22.9
		573	0.5	42.5	-	-	-	-	43.0
		623	2.3	55.0	-	-	-	-	57.3
9	VFe0,2Air, Т 873 K, 3hH2-, Т873 K, 1h	423	-	21.0	3.5	43.1	1.1	62.7	68.7
		473	0.6	23.3	12.0	39.4	2.5	50.6	77.8
		523	1.8	27.5	25.2	31.9	4.8	35.0	91.2
		573	2.8	29.1	35.7	27.7	4.7	27.7	100
		623	6.0	24.2	39.0	23.1	7.7	23.1	100

For the quantitative determination of the obtained reaction products, the internal standard method was used, taking into consideration the correction factors, which were determined as the tangent of the straight line’s slope [33]. The values ​​of conversion, selectivity and yields of reaction products were calculated according to the following formulas, which are presented in appendix (see Supplementary Material).

**Figure 13:**
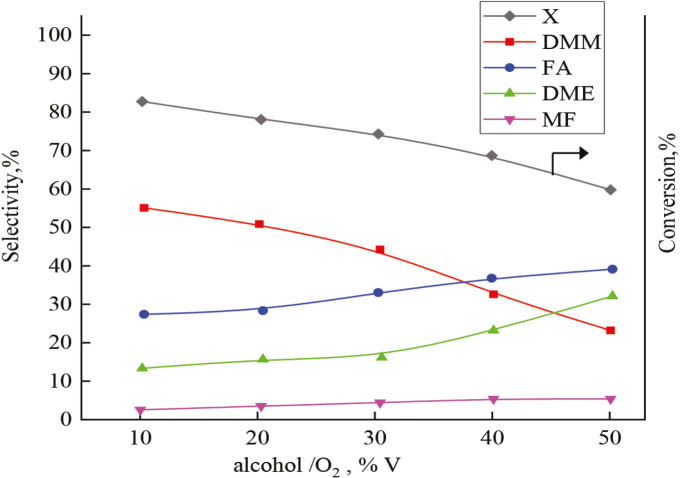
Variation of the selectivity to FA, DME, DMM with alcohol/O2 ratio on VFe 0.2 catalyst at T 473 K.

Figure 13 shows the correlation of selectivity (to FA. DMM, DME, MF), with initial concentration of alcohol (i.e., on the ratio of alcohol/O_2_) at T 473K. 

**Figure 14:**
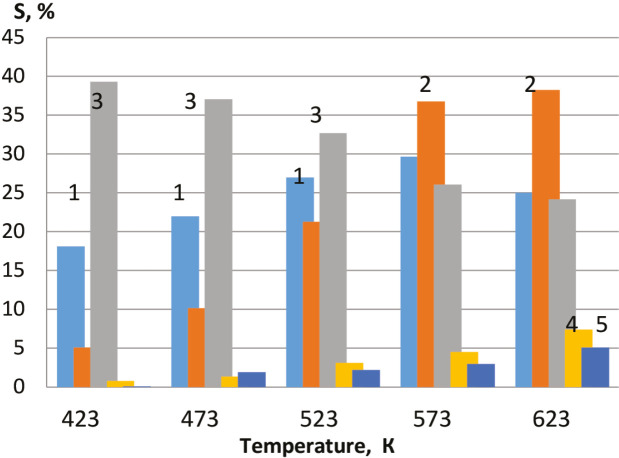
Diagram of the selectivity to FA, DME, DMM with the reaction temperature on ZrMo2 catalyst; 1–FA, 2–DME, 3–DMM, 4–MF, 5–CO2.

At a ratio of alcohol/O_2_ of 1/3, the conversion of methanol on the VFe_0.2_ catalyst after О_2_ + Н_2_-treatment was 77.8%, the selectivity for FA = 29.9%; DMM = 50.6%; DME = 15.4%, MF = 3.4%. With an increase in the alcohol: O_2_ ratio, the conversion of methanol and the selectivity for DMM at this temperature decreases, however, the selectivity for FA and DME slightly increases. A diagram at Figure 14 shows correlation of the selectivity of methanol oxidation products on ZrMo_2_ catalyst with the reaction temperature.

**Figure 15:**
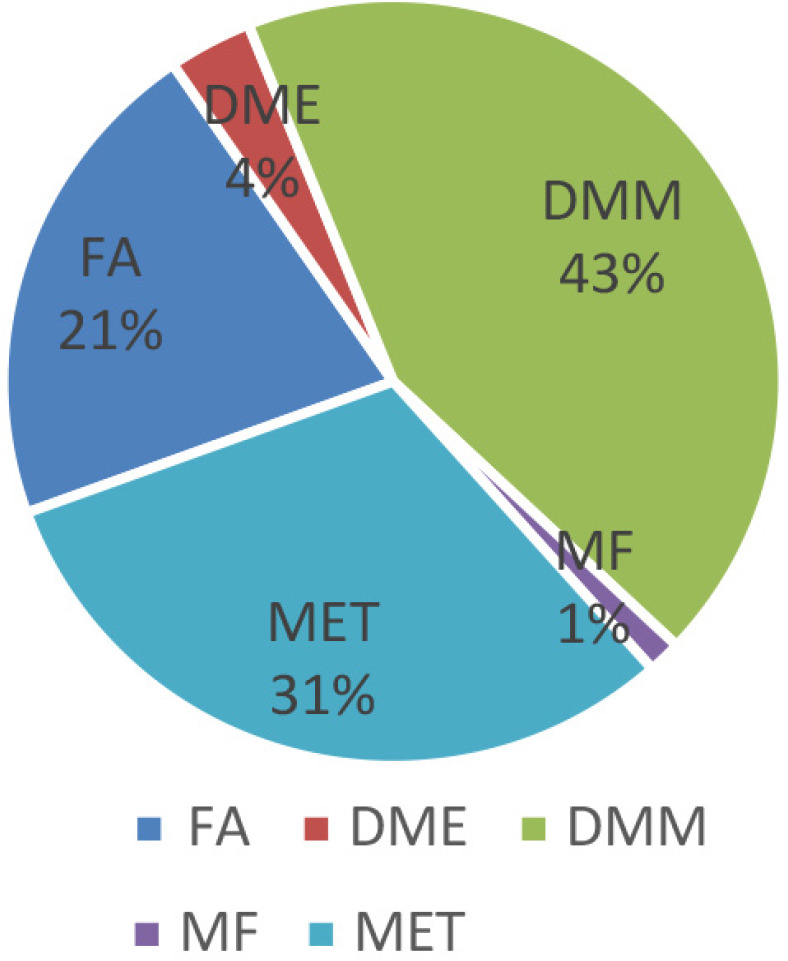
Diagram of distribution products of the methanol oxidation on VFe 0.2 catalyst at T 423 K after O2 + H2 treatment.

As seen from the Figure 14, with an increase in temperature, the selectivity for DMM decreases, while selectivity for DME, MF, and CO_2_ increases. The highest yield of DMM with a methanol conversion of 68.7% was observed on another catalyst - VFe_0.2_ after О_2 _+ Н_2_-treatment at a reaction temperature of 423 K (43%). Diagram at Figure 15 shows distribution of methanol oxidation products. According to the catalytic results, a reaction Scheme can be proposed (see Scheme 1).

Compared to the catalyst samples oxidized in a stream of air, the hydrogenated samples of the ZrV0.3; ZrMo2 and VFe0.2 alloys showed higher activity and selectivity for formaldehyde. As a result of the “soft” reductive Н2 treatment of the catalysts (Т 873 K, 1 h), a decrease in the temperature threshold of the onset of the reaction by ~ 100–150 K was observed, which is associated with the activity of the hydride subsystem at the temperature range of Т 473–523 K, which is explained in Table 3. 

## 4. Conclusion

It is proved that bimetallic catalysts based on Zr alloys with V, Mo, Fe show rather high activity and selectivity in the reaction of methanol oxidation to FA, DME, and DMM after О_2_ + Н_2_-treatment. This happens due to the segregation of the active components of alloys (V, Mo) on the surface of the catalysts in an intermediate oxidation state and, further, to the realization of their optimal oxidation state under catalysis conditions. This is evidenced by the results of XRD, XPS, and SEM analyzes.

It is assumed that the presence of V^4 +^ ↔ V^5 +^ and Mo^4 +^ ↔ Mo^5 +^ ↔ Mo^6 + ^ions and the related effect of the “electronic factor” in catalysis is enhanced in the presence of hydrogen “in situ”. It is also shown that the presence of the hydride subsystem shifts the temperature threshold of the onset of the CH_3_OH oxidation reaction towards a decrease in the reaction temperature by ~ 100–150 K. The nonstoichiometric composition of the surface layer of catalysts after О_2_ + Н_2_-treatment, the presence of anionic vacancies, the presence of a hydride subsystem, apparently, facilitated electronic transitions in bimetallic systems of Zr – V, Zr – Mo, V-Fe -catalysts. For d-elements, which included Zr, V, Mo metals, such electronic transitions were not associated with high-energy costs, and the presence of hydrogen “in situ” in these systems enhanced the influence of the “electronic factor”. The influence of these factors caused an increase in the activity of bimetallic catalysts based on Zr-V, Zr-Mo and V-Fe alloys.

Supplementary Materials
