# Supplementary material for: Feature of catalysis on bimetallic alloys Zr with V, Mo, and Fe in the reaction of methanol oxidation
Source: Turk J Chem. 2021 Aug 27;45(4):1070–85. doi: 10.3906/kim-2010-9 (PMC8517494; doi:10.3906/kim-2010-9)
Supplement: Supplementary file 1 — Supplementary Materials [file turkjchem-45-1070-sup001.pdf]

Supplementary material

Figure 1S. Scheme of a flow-through installation.

1–vent scrubbing system, 2 – flow meter, 3 – traps, 4 – saturator, 5 – mixer, 6 – reactor, 7 – six-way valve, 8 – two - way valve, 9 – three - way valve, 10 – thermostat, 11 – heating furnace, 12 – condenser.

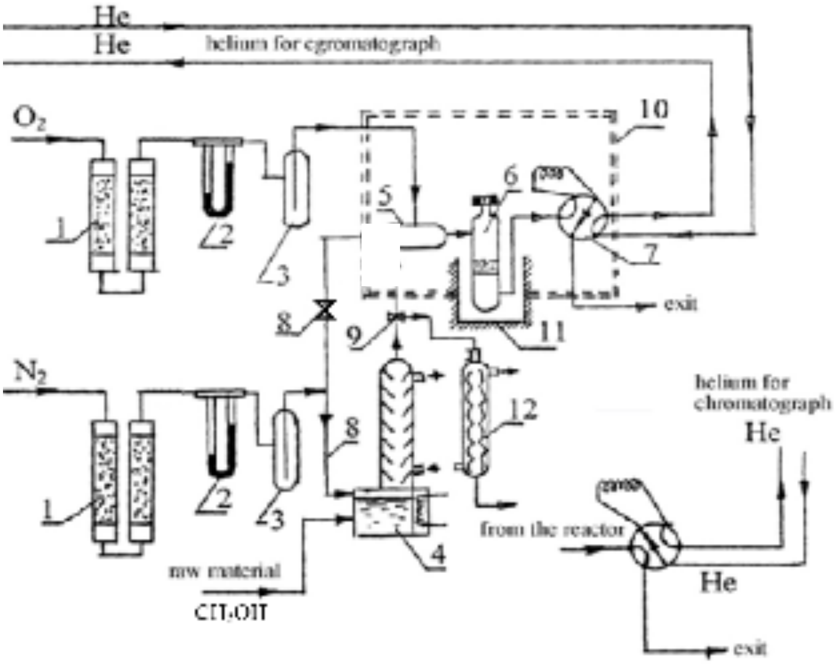

Table 1. XRD analysis outcomes for the initial $ZrV_{0.3}$.

	Experimental data						Reference data						Filling cabinet
№	2θ	d, Å	I (a.u)	h	K	l	2θ	d, Å	I (a.u)	h	k	l	JCPDS
1	32.026	2.792	100	1	0	0	31.60	2.960	100	1	1	1	
2	35.682	2.514	29.5	0	0	2	35.31	2.540	25	2	0	0	
3	37.762	2.380	15.4	1	0	0	50.15	1.830	65	2	0	2	37-1484
4	40.092	2.247	12.9	2	1	1	55.42	1.810	35	2	2	0	m- ZrO_2
5	51.592	1.770	12.5	1	0	2	60.35	1.547	45	3	1	1	
6	54.952	1.670	18.8	1	1	0	62.82	1.493	12	2	2	2	
7	62.260	1.490	29.4	1	0	3							
8	67.552	1.386	20.6	-	-	-	30.16	2.930	100	1	1	1	
							35.19	2.550	25	2	0	0	27-997
							50.65	1.801	50	2	0	2	t-ZrO_2
							60.34	1.534	20	3	1	1	
							68.82	1.471	5	2	2	2	

Table 2. XRD analysis outcomes for $ZrV_{0.3}$ after O_2- and H_2-treatment.

№	Experimental data						Filling cabinet
	2θ	d, Å	I (a.u)	H	K	L	JCPDS
$ZrV_{0.3}$ after O_2-treatment							
1	20.010	4.433	13.8	1	0	1	41- 1426
2	22.410	3.964	14.5	1	1	0	o -V_2O_5
3	23.764	3.741	20.0	1	0	2	
4	24.615	3.614	8.9	0	1	1	
5	27.968	3.187	100	1	1	1	79-1976
6	31.171	2.867	47.6	1	1	1	t-V_2O_5
7	32.155	2.782	44.3	1	0	0	
8	33.683	2.659	24.9	0	0	2	
9	35.105	2.554	7.5	2	0	0	34-0187
10	40.514	2.225	18.8	2	1	1	V_2O_3
11	44.702	2.026	12.5	2	0	2	
12	50.106	1.819	46.1	2	2	0	
13	54.142	1.692	9.4	2	0	2	
14	55.064	1.667	23.5	0	1	3	
15	62.440	1.486	17.6	2	1	3	
$ZrV_{0.3}$ after H_2-treatment							
16	21.2	2.723	11	1	1	0	
17	31.0	2.870	27.9	0	1	1	43-1051
18	32,4	2.795	100	1	0	1	VO_2
19	35.3	2.546	38.9	0	0	2	
20	41.5	2.178	73.7	2	0	1	34-0187
21	42.3	2.133	26.1	2	0	0	V_2O_3
22	44.9	2.011	19.3	2	0	2	

Table 3. XRD analysis outcomes for the $ZrMo_2$ after O_2-treatment.

№	Experimental data						Reference data						Filling cabinet
	2θ	d, Å	I (a.u)	h	k	l	2θ	d, Å	I (a.u)	h	k	L	JCPDS
Initial $ZrMo_2$													
1	32.042	2.793	100	1	0	0	31.61	2.96	100	1	1	1	
2	35.821	2.511	29.7	0	0	2	35.31	2.54	25	2	0	0	37-1484
3	37.753	2.383	15.4	1	0	0	50.15	1.83	65	2	0	2	m-ZrO_2
4	40.081	2.249	12.9	2	1	1	55.42	1.81	35	2	2	0	
5	49.560	1.839	12.5	1	0	2	60.35	1.55	46	3	1	2	
6	54.952	1.671	18.6	1	1	0							
7	62.255	1.491	28,7	1	0	3	30.16	2.93	100	1	1	1	27- 997
8	68.105	1.377	21.0				35.19	2.55	25	2	0	0	t- ZrO
							50.65	1.80	50	2	0	2	
							60.34	1.53	20	3	1	1	
$ZrMo_2$ after O_2-treatment													
9	15.132	5.850	2	0	0	2	-	-	-	-	-	-	
10	23.180	3.834	100	1	1	2	23.13	3.84	100	1	1	2	21-1496
11	30.526	2.926	41	3	0	0	30.47	2.93	47	3	0	0	$Zr(MoO_4)$
12	35.379	2.535	19	2	2	0	50.06	1.82	31	4	1	2	
13	38.661	2.327	10	2	2	2	-	-	-	-	-	-	
14	47.389	1.917	14	4	1	0	23.3	3.81	40	1	1	0	
15	50.033	1.822	23	1	1	6	25.9	3.44	100	0	4	0	76-1003
16	56.652	1.624	9	3	3	2	27.8	3.21	18	0	1	1	MoO_3
17	57.494	1.602	8	5	0	3	33.3	2.69	11	0	2	1	
18	63.501	1.463	8	6	0	0	39.1	2.28	18	1	3	1	

Table 4. XRD analysis outcomes for the $ZrMo_2$ after H_2-treatment.

№	Experimental data						Filling cabinet
	2θ	d, Å	I (a.u)	H	k	l	JCPDS
1	15.646	5.725	100	2	0	0	
2	21.034	4.220	87	2	0	2	$H_6Mo_2O_{11}Zr$
3	24.565	3.621	71.3	3	1	0	
4	28.471	3.132	84	3	1	2	13-142
5	28.563	3.125	57	0	0	4	i-Mo_4O_{11}
6	34.437	2.602	24	4	0	2	
7	36.230	2.477	18.2	3	3	2	12-753
8	38.022	2.365	11.8	4	2	2	Mo_9O_{26}
9	47.612	1.908	27.3	6	0	0	
10	48.561	1.873	14.3	5	3	2	
11	50.533	1.805	15.8	3	1	6	01-1208
12	56.465	1.628	17.2	6	0	4	Mo (met)
13	68.648	1.366	50.0	5	2	7	
14	76.904	1.238	71.0	9	1	2	
15	79.203	1.208	42.0	6	0	8	

Table 5. XRD analysis outcomes for $VFe_{0.2}$ after O_2- and H_2-treatment.

№	Experimental data						Reference data			Filling cabinet
	2θ	d, Å	I (a.u)	H	k	L	2θ	d, Å	I (a.u)	JCPDS
Initial $VFe_{0,2}$										
1	35.1	2.550	48	1	1	1	23.6	3.775	30	
2	41.3	2.181	100	1	1	0	32.7	2.730	100	34-0187
3	42.2	2.139	18.8	2	1	2	37.6	2.391	80	V_2O_3
4	44.7	2.026	37	0	1	2	41.8	2.157	20	
5	62.6	1.484	10	2	0	2	53.9	1.700	90	
$VFe_{0,2}$ after O_2-treatment										
1	20.6	4.302	37	0	0	1	30.0	1.194	100	79-1976
2	24.6	3.615	32	0	1	1	50.7	1.799	80	t- V_2O_5
3	27.0	3.305	100	1	1	0	61.0	1.516	35	
4	32.5	2.751	85	0	3	1				
5	37.4	2.398	50	1	2	1	20.3	4.375	90	
6	41.4	2.175	20	1	3	0	26,6	3.348	100	41-1426
7	47.0	1.935	48	1	4	1	31.2	2.862	70	o-V_2O_5
8	50.2	1.816	70	2	2	0	34.3	2.610	50	
9	61.5	1.504	40	1	0	4	47.3	1.920	20	
10	67.2	1.392	25	1	1	3				
$VFe_{0,2}$ after H_2-treatment										
1	21.0	4.226	5	-	-	-	26.3	3.371	100	
2	22.0	2.056	10	-	-	-	37.1	2.421	60	43-1051
3	31.0	2.882	31.7	-	-	-	42.0	2.150	15	VO_2
4	32.0	2.794	100	-	-	-	56.5	1.628	50	
5	44.5	2.029	46.1	-	-	-				
6	48.0	1.893	20.9	-	-	-	33.3	2.69	100	24-0072
7	52.5	1.738	39.1	-	-	-	35.7	2.51	80	α-Fe_2O_3
8	54.6	1.679	15.2	-	-	-	54.1	1.69	51	
9	59.0	1.564	10.9	-	-	-	64.1	1.45	40	

10	67.5	1.384	7	-	-	-				
11	74.5	1.271	10.4	-	-	-	36.8	2.44	100	19-629
12	79.0	1.211	8.7	-	-	-	59.2	1.56	40	Fe_3O_4
							65.2	1.43	50	

Definition of conversion, selectivity, yield

For the quantitative determination of the obtained reaction products, the internal standard method was used, taking into consideration the correction factors, which were determined as the tangent of the straight line's slope [33]:

$$S_i / S_{st} = f(g_i / g_{st}),$$

where S_i, S_{st} - indicate areas of peaks of the analyte and the standard g_i, g_{st} - are their weight ratios. Isopropanol was adopted as the standard.

The percentage of each component was calculated using the formula:

$$C_i = \frac{S_i k_i}{\sum S_i k_i} \cdot 100\%,$$

k_i– correction factors for the reaction components.

The values of conversion, selectivity and yield presented in Table 8, were calculated according to the following formulas:

$$\text{Conversion of } CH_3OH\text{: } X,\% = \frac{C_{al}^{0} - C_{al}^{res}}{C_{al}^{0}},$$

C_{al}^{res} –alcohol residue

$$\text{Selectivity of i – product: } S_i,\% = \frac{C_i}{\sum C_i},$$

Yield of i– product: W_i, % = X × S_i.
